# Permanent pacemaker implantation in a hemodialysis patient with subclavian vein occlusion using the balloon‐target puncture technique

**DOI:** 10.1002/ccr3.4144

**Published:** 2021-05-04

**Authors:** Yuhei Kasai, Sandeep Shakya, Takashi Yamaura, Naoki Hayakawa, Kotaro Miyaji, Junji Kanda

**Affiliations:** ^1^ Department of Cardiology Asahi General Hospital Chiba Japan

**Keywords:** balloon‐target puncture technique, carbon dioxide digital subtraction angiography, hemodialysis, permanent pacemaker implantation, subclavian vein occlusion

## Abstract

The balloon‐target puncture technique is an effective method to secure the subclavian vein access route of cardiovascular implantable electronic devices.

## Graphical abstract

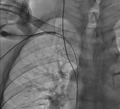

## 1 INTRODUCTION

We describe a case of permanent pacemaker implantation in a 59‐year‐old man with subclavian vein occlusion undergoing hemodialysis. The balloon‐target puncture technique aided successful and safe transvenous permanent pacemaker implantation.

The most widely used method for permanent pacemaker implantation (PMI) is via the subclavian vein. In patients undergoing hemodialysis, subclavian vein occlusion is common. A previous report described that venous stenosis or occlusion is observed in 10% of patients undergoing hemodialysis with no history of catheter placement.^1^ In such patients, permanent PMI can be challenging; thus, endovascular treatment (EVT) may be necessary.

Herein, we describe a case of EVT for subclavian vein occlusion and permanent PMI in a 59‐year‐old man undergoing hemodialysis. EVT was performed using the balloon‐target puncture technique as an alternative technique for lead implantation. To the best of our knowledge, this is the first report of balloon‐target puncture as an alternative technique for lead implantation.

## 2 CASE REPORT

The patient was a 59‐year‐old man with a 12‐year history of hemodialysis due to diabetic kidney disease. The patient had a history of failed bilateral arteriovenous fistula and underwent placement of an arteriovenous graft in his left arm 2 years ago for hemodialysis. The patient experienced syncope during hemodialysis. Electrocardiography showed complete atrioventricular block and was transported to our hospital for further treatment. Temporary PMI was immediately performed via the right internal jugular vein, and the patient was admitted for permanent PMI the following day.

Permanent PMI was planned in the right chest because of presence of the graft in the left arm. Right subclavian venography was not performed because the patient had experienced an allergic reaction to iodinated contrast 2 years prior. Instead, we decided to introduce a 0.035‐inch guidewire into the right subclavian vein (SCV) for puncture.

**FIGURE 1:**
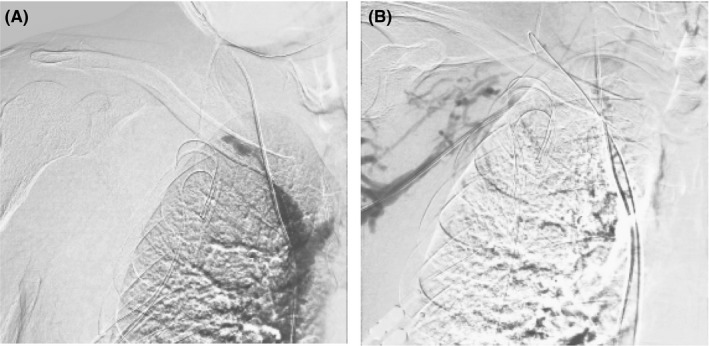
A, CO_2_‐DSA performed at the right brachiocephalic vein. CO_2_‐DSA, carbon dioxide digital subtraction angiography. B, CO_2_‐DSA performed at the distal end of the subclavian vein. CO_2_‐DSA, carbon dioxide digital subtraction angiography

During the procedure, a 5‐Fr sheath was inserted from the right femoral vein. A 0.035‐inch Radifocus wire^®^ (Terumo) with the support of a 4‐Fr Judkins right (JR) catheter^®^ (Medikit) was introduced. However, we could not pass the guidewire to the right SCV. Thus, SCV occlusion was suspected. Carbon dioxide digital subtraction angiography (CO_2_‐DSA) was performed using the JR catheter at the right brachiocephalic vein (Figure 1A), which showed total occlusion of the right SCV. To determine occlusion length, venography from the basilic vein was performed. A 5‐Fr sheath was inserted into the right basilic vein using ultrasound‐guided puncture. The 0.035‐inch Radifocus wire was inserted with the support of a 4‐Fr Glidecath^®^ (Terumo). The guidewire successfully passed to the distal end of the SCV, and CO_2_‐DSA was performed (Figure 1B).

Since the patient had a graft in the left arm and a prior history of failed bilateral arteriovenous fistula, a transvenous pacemaker was implanted on the right side. A leadless pacemaker was an alternative option, but because of a prior allergic reaction to iodinated contrast, there was a risk of developing an allergic reaction. The occlusion length was short, so EVT to the right SCV was decided.

A 0.014‐inch guidewire (Gladius^®^ Mongo^®^ PV ES; Asahi Intecc) with the support of a 4‐Fr Glidecath was introduced via the right basilic vein and penetrated the occlusion successfully before being advanced into the superior vena cava. A 5.0 × 40‐mm Mustang^®^ balloon (Boston Scientific) could not pass the occlusion. We predilated the lesion using a 2.0 × 40‐mm Coyote^®^ followed by a 4.0 × 40‐mm Coyote (Boston Scientific). Using the Glidecath, we changed the Gladius Mongo PV ES to a 0.035‐inch Radifocus wire. The 5.0 × 40‐mm Mustang passed the lesion, and balloon angioplasty was performed. Then, we used a 21‐G micro‐puncture needle (Cook) to puncture the right SCV. However, puncture was not successful as venous blood could not be drawn. Vein elastic recoil was considered, and we decided to puncture the balloon instead.

**FIGURE 2:**
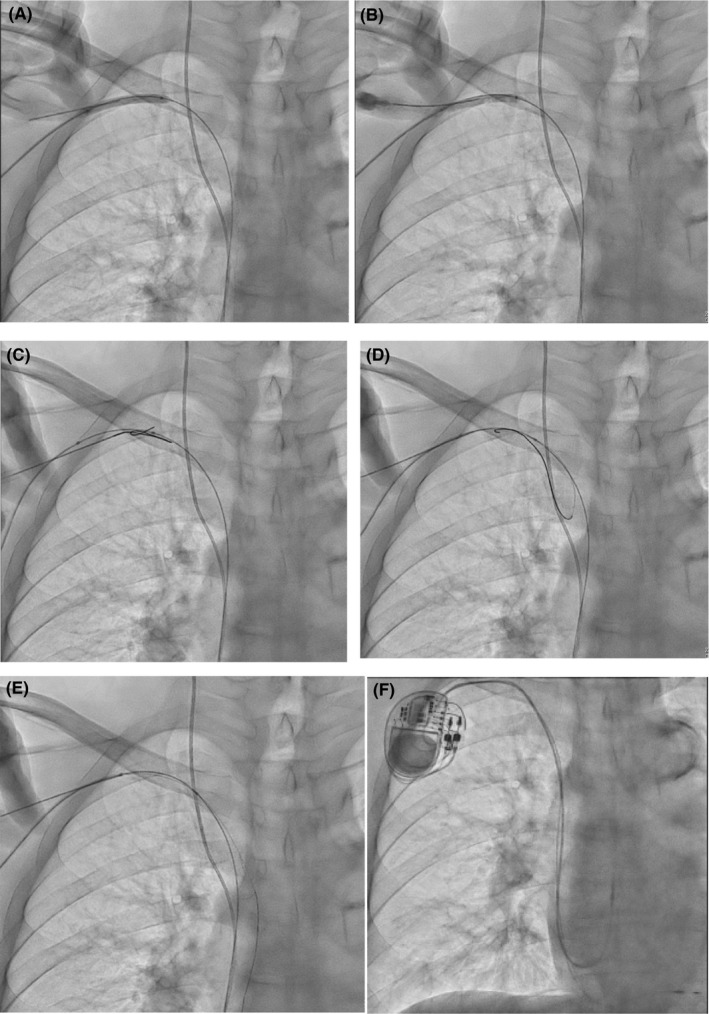
A, When a 5.0 × 40‐mm Mustang was dilated in the SCV, we attempted to puncture the balloon with a micro‐puncture needle. SCV, subclavian vein. B, Because of balloon puncture, the diluted contrast in the balloon was aspirated into the syringe, which revealed that the micro‐puncture needle tip was in the SCV. SCV, subclavian vein. C, D, Both the balloon and guidewire were pushed. The guidewire was advanced in the same way as a knuckle wire technique in the same way as a knuckle wire technique. E, The guidewire was successfully advanced into the SCV. SCV, subclavian vein. F, A dual‐chamber pacemaker was implanted. The atrial lead was implanted into the right atrial appendage, and the ventricular lead was implanted into the right ventricular septum

The 5.0 × 40‐mm Mustang was dilated in the SCV. Using a 21‐G micro‐puncture needle attached to a 2.5‐mL syringe, we punctured the balloon under the guidance of fluoroscopy. On successful puncture, almost all diluted contrasts were aspirated into the syringe (Figure 2A,B). The guidewire was inserted; however, the guidewire alone could not pass into the vein, and both the balloon and guidewire were pushed. Subsequently, the guidewire was successfully advanced into the SCV (Figure 2C,D,E). This sequence of procedures is named the balloon‐target puncture technique.

We used the one‐puncture two‐sheath technique to introduce two 7‐Fr peel‐off sheaths (Medikit) into the SCV and successfully implanted the pacemaker (Figure 2F). The SelectSecure^™^ MRI SureScan^™^ lead (Medtronic) was implanted using a C315 S10 guiding catheter into the right ventricular septum, and the CapSureFix Novus MRI^™^ SureScan screw‐in lead was implanted into the right atrial appendage. The procedural duration was 2 hours 25 minutes, and the fluoroscopy time was 36 minutes. No perioperative complications occurred. The patient was discharged after 1 week.

## 3 DISCUSSION

The subclavian route is commonly used for insertion of cardiovascular implantable electronic devices. However, in the presence of subclavian vein occlusion, this route cannot be used. To circumvent this issue, a number of venous system interventions for cardiovascular implantable electronic device implantation have been reported.^2^, ^3^ However, to the best of our knowledge, there have been no reports on how to puncture the SCV safely after SCV recanalization. Herein, the balloon‐target puncture technique, which can achieve safe puncture, was used in a 59‐year‐old man undergoing hemodialysis with SCV occlusion.

This technique has some advantages. First, it is effective in cases of elastic recoil after balloon angioplasty. In this case, we tried to puncture the SCV using a 21‐G micro‐puncture needle toward the 0.035‐inch Radifocus, but this method was unsuccessful despite three attempts at puncture. At first, the 5.0 × 40‐mm Mustang balloon could not pass the occlusion. Subsequently, we predilated the lesion using a 2.0 × 40‐mm Coyote followed by a 4.0 × 40‐mm Coyote using a method termed stepwise balloon angioplasty. Despite stepwise balloon angioplasty, it remained difficult to pass a 5.0 × 40‐mm Mustang through the lesion, which suggested elastic recoil of the puncture lesion after balloon angioplasty. Therefore, puncturing the dilated balloon of the elastic recoiled lesion was a reasonable approach to take.

Second, this technique makes it possible to puncture all regions of the SCV away from the ribcage. Normally, we puncture the SCV above the ribcage to prevent pneumothorax. With this technique, the need for the Seldinger method is significantly reduced. Therefore, we do not necessarily have to puncture the SCV above the ribcage.

Third, this technique suggests that we should not puncture lesions refractory to balloon dilatation. Jose et al reported that approximately 3% of patients who require EVT for the SCV have a lesion refractory to an ultra‐noncompliant balloon (rated balloon pressure: 30 atm).^4^ In this case, fortunately, there were no SCV lesions refractory to noncompliant balloon dilatation. If possible, we should puncture proximal to the lesion refractory to balloon dilatation to reduce damage to cardiovascular implantable electronic device leads.

Finally, this technique can be used in patients who require addition or replacement of cardiovascular implantable electronic device leads. The incidence of SCV stenosis or occlusion after device implantation varies widely in the literature (ranging from 25% to 50%^5^, ^6^, ^7^, ^8^); however, situations in which this technique should be used are not uncommon.

To perform the balloon‐target puncture technique, we need no additional balloons and catheters for patients with transvenous cardiovascular implantable electronic devices who require EVT. When SCV puncture using conventional puncture methods, such as guidewire or contrast‐guided methods, is difficult, the balloon‐target puncture technique should be actively used as a cost‐effective option.

## 4 CONCLUSION

The balloon‐target puncture technique could be useful during intravenous permanent PMI requiring SCV intervention.

## CONFLICT OF INTEREST

None declared.

## AUTHOR CONTRIBUTIONS

YK: wrote the manuscript and was the second EVT operator and the first PMI operator. SS: supervised the writing of the manuscript and was the first EVT operator and the second PMI operator. TY: assisted both EVT and PMI. NH: supervised the EVT procedure. KM: supervised the PMI procedure. JK: supervised the project. All authors: read and approved the final manuscript.

## ETHICAL APPROVAL

The enrolled patient provided written informed consent. The examination was made in accordance with the approved principles. All the preparations and the equipment used are officially certified for the clinical use.

## Data Availability

Data are available on request.
